# Fractional Flow Reserve/Instantaneous Wave-Free Ratio Discordance in Angiographically Intermediate Coronary Stenoses: An Analysis Using Doppler-Derived Coronary Flow Measurements

**DOI:** 10.1016/j.jcin.2017.09.021

**Published:** 2017-12-26

**Authors:** Christopher M. Cook, Allen Jeremias, Ricardo Petraco, Sayan Sen, Sukhjinder Nijjer, Matthew J. Shun-Shin, Yousif Ahmad, Guus de Waard, Tim van de Hoef, Mauro Echavarria-Pinto, Martijn van Lavieren, Rasha Al Lamee, Yuetsu Kikuta, Yasutsugu Shiono, Ashesh Buch, Martijn Meuwissen, Ibrahim Danad, Paul Knaapen, Akiko Maehara, Bon-Kwon Koo, Gary S. Mintz, Javier Escaned, Gregg W. Stone, Darrel P. Francis, Jamil Mayet, Jan J. Piek, Niels van Royen, Justin E. Davies

**Affiliations:** a Imperial College London, London, United Kingdom; b St. Francis Hospital, Roslyn, New York; c Cardiovascular Research Foundation, New York, New York; d VU University Medical Centre, Amsterdam, the Netherlands; e Academic Medical Centre, Amsterdam, the Netherlands; f Instituto de Seguridad y Servicios Sociales de los Trabajadores del Estado, Mexico City; g East Carolina Heart Institute at East Carolina University, Greenville, North Carolina; h Amphia Hospital, Breda, the Netherlands; i Department of Medicine, Columbia University Medical Center, New York, New York; j Seoul National University Hospital, Seoul, Republic of Korea; k Cardiovascular Institute, Hospital Clínico San Carlos, Madrid, Spain

**Keywords:** CFR, coronary flow reserve, coronary physiology, FFR, fractional flow reserve, iFR, instantaneous wave-free ratio, CFR, coronary flow reserve, FFR, fractional flow reserve, iFR, instantaneous wave-free ratio, IQR, interquartile range, MACE, major adverse cardiac events, Pa, aortic pressure, Pd, distal coronary pressure

## Abstract

**Objectives:**

The study sought to determine the coronary flow characteristics of angiographically intermediate stenoses classified as discordant by fractional flow reserve (FFR) and instantaneous wave-free ratio (iFR).

**Background:**

Discordance between FFR and iFR occurs in up to 20% of cases. No comparisons have been reported between the coronary flow characteristics of FFR/iFR discordant and angiographically unobstructed vessels.

**Methods:**

Baseline and hyperemic coronary flow velocity and coronary flow reserve (CFR) were compared across 5 vessel groups: FFR+/iFR+ (108 vessels, n = 91), FFR–/iFR+ (28 vessels, n = 24), FFR+/iFR– (22 vessels, n = 22), FFR–/iFR– (208 vessels, n = 154), and an unobstructed vessel group (201 vessels, n = 153), in a post hoc analysis of the largest combined pressure and Doppler flow velocity registry (IDEAL [Iberian-Dutch-English] collaborators study).

**Results:**

FFR disagreed with iFR in 14% (50 of 366). Baseline flow velocity was similar across all 5 vessel groups, including the unobstructed vessel group (p = 0.34 for variance). In FFR+/iFR– discordants, hyperemic flow velocity and CFR were similar to both FFR–/iFR– and unobstructed groups; 37.6 (interquartile range [IQR]: 26.1 to 50.4) cm/s vs. 40.0 [IQR: 29.7 to 52.3] cm/s and 42.2 [IQR: 33.8 to 53.2] cm/s and CFR 2.36 [IQR: 1.93 to 2.81] vs. 2.41 [IQR: 1.84 to 2.94] and 2.50 [IQR: 2.11 to 3.17], respectively (p > 0.05 for all). In FFR–/iFR+ discordants, hyperemic flow velocity, and CFR were similar to the FFR+/iFR+ group; 28.2 (IQR: 20.5 to 39.7) cm/s versus 23.5 (IQR: 16.4 to 34.9) cm/s and CFR 1.44 (IQR: 1.29 to 1.85) versus 1.39 (IQR: 1.06 to 1.88), respectively (p > 0.05 for all).

**Conclusions:**

FFR/iFR disagreement was explained by differences in hyperemic coronary flow velocity. Furthermore, coronary stenoses classified as FFR+/iFR– demonstrated similar coronary flow characteristics to angiographically unobstructed vessels.

In determining the physiological significance of an angiographically intermediate coronary stenosis, the fractional flow reserve (FFR) and instantaneous wave-free ratio (iFR) both quantify the trans-stenotic pressure ratio as a surrogate measure of coronary flow. FFR is measured under conditions of maximal pharmacological hyperemia (1) whereas iFR is measured in the resting state (2).

In up to 20% of cases, FFR and iFR disagree on the functional significance of a stenosis (3). The recently reported DEFINE-FLAIR (Functional Lesion Assessment of Intermediate Stenosis to Guide Revascularisation) (4) and iFR-SWEDEHEART (Evaluation of iFR vs FFR in Stable Angina or Acute Coronary Syndrome) (5) trials demonstrated in over 4,500 patients that iFR was noninferior to revascularization guided by FFR with respect to major adverse cardiac events (MACE) at 1 year. Furthermore, patient-level pooled meta-analysis of both trials demonstrated significantly less revascularization based on iFR versus FFR interrogation, but similar MACE in the both FFR and iFR deferred populations (6). This combination of findings have lead some to question whether, in comparison to iFR, FFR overestimates the true flow-limiting potential of angiographically intermediate coronary stenoses.

In this study we performed a dedicated post hoc analysis of stenosis classification discordance between FFR and iFR using combined coronary pressure-and-flow measurements from the multicenter Iberian-Dutch-English (IDEAL) collaborators registry on coronary physiology (7). The aim of this study was to determine the coronary flow characteristics of angiographically intermediate stenoses classified as discordant by FFR and iFR with comparison to an angiographically unobstructed vessel group.

## Methods

### Study population

This post hoc, retrospective analysis included a total of 567 vessels (n = 301), comprising 366 stenosed vessels (n = 291) and 201 unobstructed vessels (n = 153), as part of the Iberian–Dutch–English collaborators (IDEAL study) study dataset (7). The IDEAL study is the largest international, multicenter, nonrandomized, prospective analysis in patients with coronary artery disease undergoing physiological lesion assessment by combined pressure (FFR and iFR) and Doppler flow velocity measurements. All patients recruited were scheduled for elective coronary angiography with physiological stenosis assessment by FFR and gave written informed consent for acquisition of additional physiological data for study purposes. Stenosed vessels were defined as vessels that had an angiographically visible stenosis between 40% to 70% severity, as determined visually by the operating physician at the time of coronary angiography. Unobstructed vessels were defined as vessels with a complete absence of any angiographically visible stenosis. As part of the original IDEAL study protocol, all angiogram cines were reviewed and adjudicated by 2 independent assessors to ensure compliance with the aforementioned definitions (7).

Exclusion criteria were limited to severe valvular heart disease, acute myocardial infarction within 48 h, previous coronary artery bypass surgery, vessels with angiographically identifiable myocardial bridging or collateral arteries, and vessels with a previous myocardial infarction.

### Coronary catheterization and measurement of physiologic indices

Physiological measurements of coronary stenoses were performed according to the existing IDEAL study protocol (7). Briefly, for pressure-based measurements the pressure sensor was first zeroed and equalized to aortic pressure, before being positioned at least 3 vessel diameters distal to the stenosis and a recording of the baseline distal coronary and aortic pressures obtained. Adenosine was administered by intravenous infusion in 234 measurements (140 μg/kg/min) and by intracoronary bolus injection in 333 measurements (60 to 150 μg).

FFR was calculated as the ratio of mean distal coronary artery pressure to mean aortic pressure across the whole cardiac cycle during hyperemia. iFR was calculated as the mean pressure distal to the stenosis divided by the mean aortic pressure during the wave-free period of diastole.

Intracoronary nitrates (200 to 300 μg) were administered in all cases before performing any physiological measurement. Resting indices were calculated at a time of stability, allowing for a return to stable baseline conditions after any preceding injection of contrast or saline. Hyperemic indices were calculated during stable hyperemia, excluding ectopy and conduction delay.

Significant drift was defined as ±2 mm Hg (8) after pullback of the pressure wire transducer into the guiding catheter. If pressure drift was identified, measurements were repeated or corrected for on analysis.

For flow-based measurements, Doppler signals were optimized carefully to ensure adequate tracking profiles were observed. Electrocardiography, pressures, and flow velocity signals were directly extracted from the device console (ComboMap, Volcano Corporation, San Diego, California). Data were analyzed offline, using a custom software package designed with MATLAB version 6.0.0.88 (The MathWorks, Natick, Massachusetts). The calculations for the physiology indices used in the study are shown in Table 1.

**Table 1:** Definition of Physiological Indices

Pa	Proximal (aortic) pressure (mm Hg)
Pd	Distal (coronary) pressure (mm Hg)
FFR	Pd/Pa at whole-cycle hyperemia
iFR	Pd/Pa at baseline iFR window
Baseline coronary flow velocity	Mean baseline whole-cycle coronary flow velocity (cm/s)
Hyperemic coronary flow velocity	Mean hyperemic whole-cycle coronary flow velocity (cm/s)
CFR	Whole cycle hyperemic flow velocity/Whole cycle baseline flow velocity

CFR = coronary flow reserve; FFR = fractional flow reserve; iFR = instantaneous wave-free ratio; Pa = aortic pressure; Pd = distal coronary pressure.

### Comparison of coronary flow characteristics between groups

Established cutoff values of pressure-derived physiologic indices (FFR ≤0.80 and iFR ≤0.89) 9, 10 were used to dichotomize stenoses into concordantly classified (FFR+/iFR+ and FFR–/iFR–) and discordantly classified (FFR+/iFR– and FFR–/iFR+) groups. Baseline coronary flow velocity (cm/s), hyperemic coronary flow velocity (cm/s), and coronary flow reserve (CFR) were compared across these groups, as well as in the unobstructed vessel group.

### Statistical analysis

Categorical data were expressed as numbers and percentages, while continuous data were expressed as mean ± SD or median (interquartile range [IQR]) as appropriate. Tests of normality were first performed using the Shapiro-Wilk test. Continuous variables were compared with Student t or Mann-Whitney *U* tests, and categorical variables with chi-square or Fisher exact tests, as appropriate. Differences across the groups were compared with the Kruskal-Wallis *H* test, followed by post hoc Mann-Whitney *U* tests with Bonferroni correction. Cohen’s kappa coefficient was used to assess agreement between dichotomous variables. Applicable tests were 2 tailed and p < 0.05 was considered statistically significant. All analyses were performed using R version 3.2.1 (R Project for Statistical Computing, Vienna, Austria).

## Results

### Study population

A total of 366 stenosed vessels and 201 unobstructed vessels were derived from a total study population of 301 patients (60.6 ± 9.6 years of age, 69% men) (Table 2). The patient characteristics of the FFR/iFR discordant vessel groups are summarized in Table 3. In comparison to the FFR+/iFR– group, the FFR–/iFR+ group demonstrated a significantly higher prevalence of diabetes (p = 0.03).

**Table 2:** Patient Demographics and Stenosis Characteristics

Patients	301
Age, yrs	60.6 ± 9.6
Male	209 (69)
Hypertension	157 (52)
Hyperlipidemia	172 (57)
Current or ex-smoker	128 (43)
Diabetes mellitus	67 (22)
Chronic renal impairment	5 (2)
Family history of CAD	129 (43)
Previous myocardial infarction	34 (11)
Impaired LV function EF <30%	2 (0.7)
Stable angina	290 (96)
Unstable angina	11 (4)
Vessels	567
Angiographically stenosed vessels	366
Patients contributing 1 vessel	228/291 (78)
Patients contributing 2 vessels	51/291 (18)
Patients contributing 3 vessels	12/291 (4)
Angiographically unobstructed vessels	201
Patients contributing 1 vessel	118/153 (77)
Patients contributing 2 vessels	22/153 (14)
Patients contributing 3 vessels	13/153 (8)
Coronary artery	
Left anterior descending	277 (49)
Left circumflex	172 (30)
Right coronary artery	118 (21)

Values are n, mean ± SD, n (%), or n/N (%).

CAD = coronary artery disease; EF = ejection fraction; LV = left ventricular.

**Table 3:** Study Population Characteristics of the FFR/iFR Discordant Vessel Groups

	FFR−/iFR+ Vessel Group (n = 24)	FFR+/iFR− Vessel Group (n = 22)	p Value
Vessels	28	22	
Patients	24	22	
Age, yrs	58.3 ± 11.1	65 ± 9.69	0.08
Male	62.5 (15)	81.8 (18)	0.15
Hypertension	58.3 (14)	50 (11)	0.57
Hypercholesterolemia	66.7 (16)	63.6 (14)	0.83
History of smoking	12.5 (3)	36.3 (8)	0.06
Diabetes mellitus	41.7 (10)	13.6 (3)	0.03∗
Chronic renal failure	0 (0)	4.5 (1)	NA
Previous MI	12.5 (3)	18.8 (4)	0.59
Family history of CVD	29.2 (7)	31.8 (7)	0.85

Values are % (n) or mean ± SD.

CVD = cardiovascular disease; MI = myocardial infarction; NA = nonapplicable; other abbreviations as in Table 1.

∗ p < 0.05.

### Stenosis and hemodynamic characteristics

**Figure 1:**
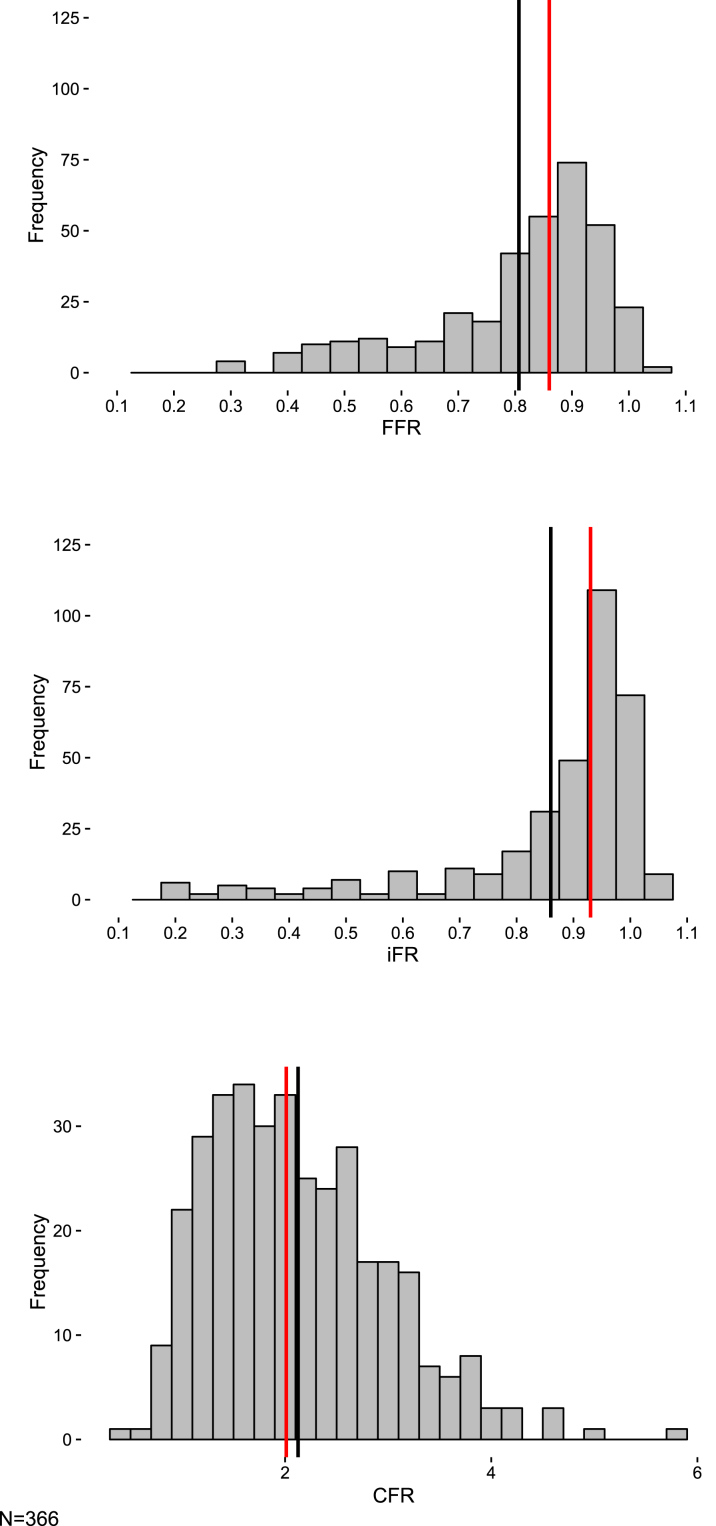
Distribution of FFR, iFR, and CFR Values for Stenosed Vessels Frequency histograms reveal unimodal data distributions of fractional flow reserve (FFR), instantaneous wave-free ratio (iFR), and coronary flow reserve (CFR) values in the stenosed vessel groups. The **solid red line** indicates the median value. The **solid black line** indicates the mean value.

The stenosis and hemodynamic characteristics of all groups are summarized in Table 4. In the stenosed vessel group, median physiological values were 0.85 (IQR: 0.74 to 0.91) for FFR, 0.93 (IQR: 0.84 to 0.97) for iFR and 1.99 (IQR: 1.44 to 2.62) for CFR. In the unobstructed group, median CFR was 2.50 (IQR: 2.11 to 3.17). The distributions of FFR, iFR, and CFR values for the stenosed vessel group are shown in Figure 1.

**Table 4:** Studied Vessel Characteristics

	FFR+/iFR+	FFR–/iFR+	FFR+/iFR–	FFR–/iFR–	Unobstructed	p Value for Variance Across Groups
Vessels	108	28	22	208	201	
Patients	91	24	22	154	153	
Stenosis characteristics						
Stenosis diameter, %	62.1 ± 17.8	48.7 ± 21.7	46.4 ± 15.8	40.0 ± 20.0		<0.01
Reference lumen diameter, mm	2.79 ± 0.9	2.81 ± 0.93	3.11 ± 0.77	2.85 ± 0.67		0.57
Minimal lumen diameter, mm	0.97 ± 0.40	1.42 ± 0.81	1.58 ± 0.61	1.67 ± 0.72		<0.01
Stenosis length, mm	19.2 ± 15.8	17.6 ± 13.1	18.9 ± 6.32	16.5 ± 12.5		0.54
Hemodynamics						
Resting heart rate, beats/min	79 ± 24	72 ± 11	73 ± 17	75 ± 18	76 ± 21	0.25
Baseline Pa, mm Hg	98.9 ± 14.4	94.0 ± 17.7	103.0 ± 17.4	100.0 ± 14.7	98.8 ± 15.5	0.14
Baseline Pd, mm Hg	75.3 ± 18.2	85.9 ± 16.6	99 ± 18	97.8 ± 14.8	97.2 ± 15.3	<0.01
Pressure measurements						
FFR	0.63 (0.51–0.72)	0.86 (0.84–0.88)	0.77 (0.74–0.80)	0.91 (0.87–0.95)	0.97 (0.94–0.99)	
iFR	0.72 (0.50–0.84)	0.88 (0.84–0.89)	0.92 (0.91–0.93)	0.97 (0.94–0.99)	0.98 (0.96–1.00)	
Flow measurements						
Baseline flow, cm/s	16.4 (11.3–23.4)	19.3 (12.9–26.8)	15.1 (12.6–19.5)	16.9 (13.0–21.6)	16.5 (12.6–21.3)	0.34
Hyperemic flow, cm/s	23.5 (16.4–34.9)	28.2 (20.5–39.7)	37.6 (26.1–50.4)	40.0 (29.7–52.3)	42.2 (33.8–53.2)	<0.01
CFR	1.39 (1.06–1.88)	1.44 (1.29–1.85)	2.36 (1.93–2.81)	2.41 (1.84–2.94)	2.50 (2.11–3.17)	<0.01
Proportion with CFR <2, %	81.5	85.7	27.3	32.7	18.9	

Values are n, mean ± SD, or median (interquartile range).

Abbreviations as in Table 1.

### Relationships between FFR and iFR

**Figure 2:**
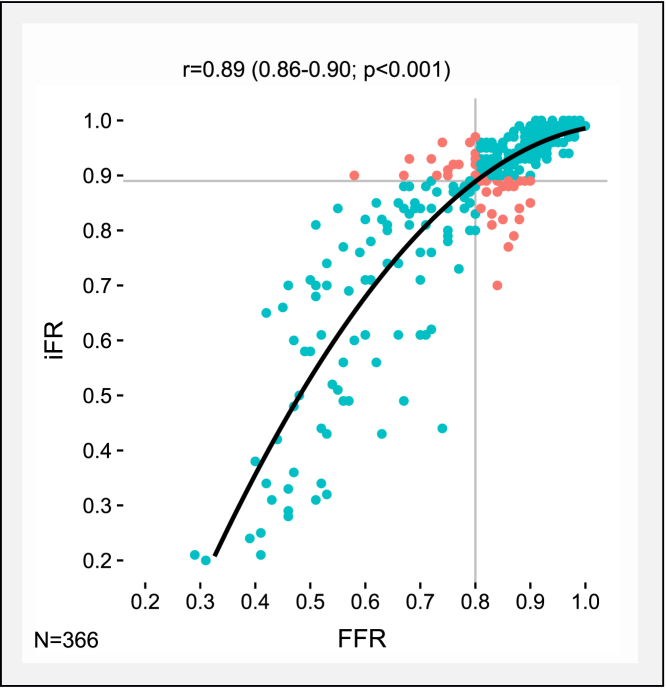
Scatter Plot Showing the Relationship Between FFR and iFR The **black line** represents the line of best fit. The curve is fitted by second-order polynomial. The **gray lines** represent the respective cutoff values for FFR (≤0.80) and iFR (≤0.89). Concordant cases are colored **blue**, discordant cases are colored **orange**. Abbreviations as in Figure 1.

Figure 2 shows the scatter plot between FFR and iFR pressure-only indices of stenosis severity. The correlation coefficient (r) between FFR versus iFR was 0.89 (95% confidence interval for the estimated correlation coefficient: 0.86 to 0.90; p < 0.001). In total, FFR agreed with iFR in 86% (316 of 366) of stenosed vessels, comprising 108 FFR+/iFR+ and 208 FFR–/iFR– cases. FFR disagreed with iFR in 14% (50 of 366) of stenosed vessels, comprising of 22 FFR+/iFR– and 28 FFR–/iFR+ discordant cases (Figure 2). Cohen’s kappa coefficient between FFR and iFR categorization was 0.71 (p < 0.001). Agreement between iFR and CFR was superior compared with the agreement between FFR and CFR, as demonstrated by a Cohen’s kappa coefficient of 0.47 (p < 0.001) versus 0.30 (p < 0.001), respectively.

### Comparisons of baseline flow velocity, hyperemic flow velocity, and CFR

**Figure 3:**
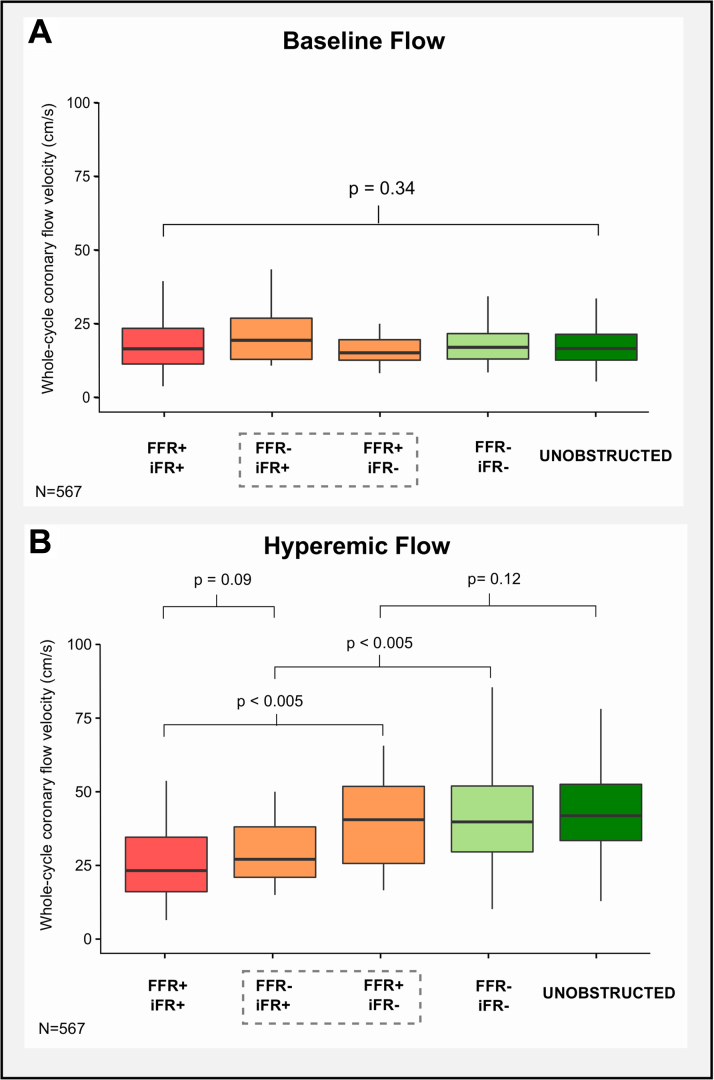
Boxplot Comparisons of Baseline and Hyperemic Coronary Flow Velocity The **horizontal black line** indicates the median value. The **box** indicates the interquartile range and the **whiskers** indicate the range of values. FFR+/iFR+ (n = 108) cases are colored **red**. FFR–/iFR+ (n = 28) and FFR+/iFR– (n = 22) discordant cases are colored **orange**. FFR–/iFR– (n = 208) cases are colored **light green**. Unobstructed reference vessel (n = 201) cases are colored **dark green**. **(A)** Baseline coronary flow velocity was similar across all groups. **(B)** Hyperemic coronary flow velocity was similar in FFR+/iFR+ and FFR–/iFR+ groups. Hyperemic coronary flow velocity was similar in FFR+/iFR–, FFR–/iFR– and unobstructed reference vessel groups. Abbreviations as in Figure 1.

Boxplots demonstrating the variations in: 1) baseline and hyperemic flow velocity; and 2) CFR according to FFR and iFR classification are shown in Figure 3 and the Central Illustration, respectively. Data from the unobstructed vessel group are also displayed.

**Central Illustration:**
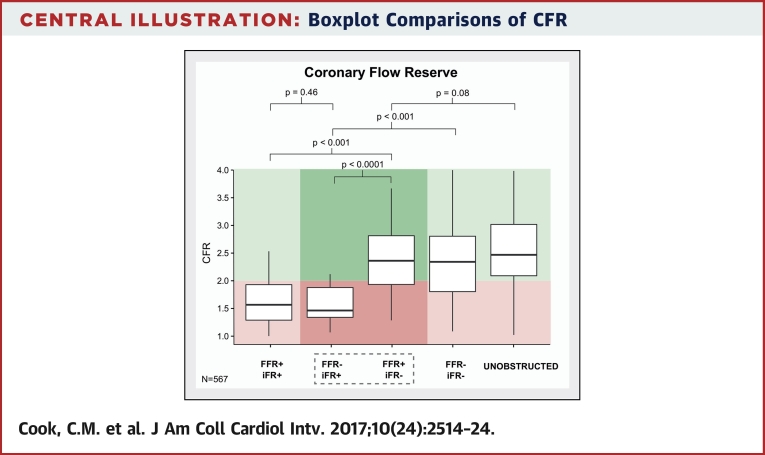
Boxplot Comparisons of CFR The **horizontal black line** indicates the median value. The **box** indicates the interquartile range, and the **whiskers** indicate the range of values. Coronary flow reserve (CFR) values ≤2 and >2 are colored **pink** and **green**, respectively. CFR was significantly higher in the fractional flow reserve (FFR) positive and instantaneous wave-free ratio (iFR) negative versus FFR–/iFR+ discordant groups (and similar to FFR–/iFR– and unobstructed reference vessel groups).

Baseline coronary flow velocity was similar across all groups (p = 0.34 for variance), with a median cross-population value of 16.6 (IQR: 12.6 to 22.06) cm/s (Figure 3A). As would be expected, hyperemic coronary flow velocity was significantly lower in FFR+/iFR+ concordantly positive versus FFR–/iFR– concordantly negative and unobstructed groups: 23.5 (IQR: 16.4 to 34.9) cm/s versus 40.0 (IQR: 29.7 to 52.3) cm/s and 42.2 (IQR: 33.8 to 53.2) cm/s, respectively (p < 0.001 for both comparisons) (Figure 3B). Similarly, CFR was significantly lower in FFR+/iFR+ concordantly positive versus FFR–/iFR– concordantly negative and unobstructed groups: CFR 1.39 (IQR: 1.06 to 1.88) versus 2.41 (IQR: 1.84 to 2.94) and 2.50 (IQR: 2.11 to 3.17), respectively (p < 0.001 for both comparisons) (Central Illustration).

For stenoses discordantly classified as positive by FFR and negative by iFR (FFR+/iFR–), no significant difference in hyperemic coronary flow velocity was observed in comparison with the FFR–/iFR– concordantly negative and unobstructed vessel groups: 37.6 (IQR: 26.1 to 50.4) cm/s versus 40.0 (IQR: 29.7 to 52.3) cm/s and 42.2 (IQR: 33.8 to 53.2) cm/s, respectively (p = 0.12) (Figure 3B). Similarly, no significant difference was found in CFR between FFR+/iFR– stenoses and FFR–/iFR– concordantly negative and unobstructed vessel groups: 2.36 (IQR: 1.93 to 2.81) versus 2.41 (IQR: 1.84 to 2.94) and 2.50 (IQR: 2.11 to 3.17), respectively (p = 0.08) (Central Illustration).

For stenoses discordantly classified as negative by FFR and positive by iFR (FFR–/iFR+), hyperemic coronary flow velocity and CFR were similar to the FFR+/iFR+ concordantly positive group: 28.2 (IQR: 20.5 to 39.7) cm/s versus 23.5 (IQR: 16.4 to 34.9) cm/s and 1.44 (IQR: 1.29 to 1.85) versus 1.39 (IQR: 1.06 to 1.88), respectively (p = 0.09 and p = 0.46, respectively).

## Discussion

The main findings of the study were as follows. First, in this cohort of angiographically intermediate stenoses, differences in stenosis classification between FFR and iFR were explained by differences in hyperemic coronary flow velocity. Second, in comparison to patients with FFR+/iFR– discordant stenoses, patients with FFR–/iFR+ discordant stenoses had a significantly higher prevalence of diabetes. Last, stenoses discordantly classified as FFR+/iFR– demonstrated similar non–flow-limiting characteristics to angiographically unobstructed vessels.

### Revascularization guided by ischemia: Flow is more important than pressure

Blood flow down the coronary arteries facilitates oxygen delivery and removal of waste metabolites from respiring myocardial cells. If this flow of blood is impeded by a coronary stenosis, supply-demand mismatch can occur, leading to myocardial ischemia and the onset of the symptoms of angina 11, 12. Positron emission tomography and stress echocardiography with Doppler assessment of coronary flow velocity all provide noninvasive measures of coronary flow. However, invasive measures of coronary flow are not routinely performed in clinical practice. Factors that contribute to this are that invasive coronary flow measurements are technically more challenging and time consuming to perform than intracoronary pressure measurements. For these reasons, despite the physiological importance of measuring intracoronary flow, the hemodynamic impact of a stenosis is most routinely assessed using pressure-based indices such as FFR and iFR.

### Relationship between coronary pressure and flow

To understand the physiological mechanisms that underpin discordance between hyperemic (FFR) and nonhyperemic (iFR) pressure-only indices of stenosis severity, combined coronary pressure-and-flow measurements are required. The relationship between pressure loss due to a stenosis (ΔP) and arterial flow velocity (V) is related by the equation, ΔP = FV + SV^2^, where F is the coefficient of pressure loss due to viscous friction in the stenotic segment and S is the coefficient of pressure loss due to flow separation at the diverging end of the stenosis (13).

**Figure 4:**
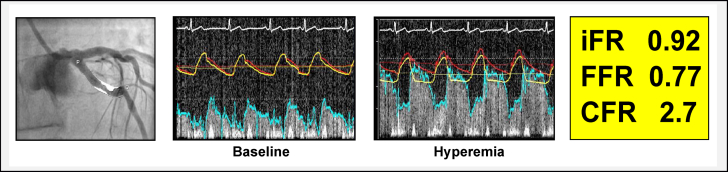
FFR+/iFR– Discordance Attributed to High CFR: Clinical Case The coronary angiogram image displays a proximal circumflex stenosis. Quantitative coronary angiography derived percentage diameter stenosis, area stenosis, and minimal lumen diameter were 62%, 85%, and 1.20 mm, respectively. Invasive pressure-based coronary physiology assessment revealed discordant iFR (negative) and FFR (positive) results. Upon measuring combined coronary pressure-and-flow data, the FFR+/iFR– discordant result can be attributed to high CFR. Abbreviations as in Figure 1.

Therefore, if arterial flow velocity (V) increases by a large amount during hyperemia, the trans-stenotic pressure gradient (ΔP) also increases. In this scenario, the resting distal coronary pressure (Pd) value falls and the resultant FFR value is low; categorizing the stenosis as functionally significant despite demonstrably high coronary flow conditions (Figure 4).

Observations regarding this form of coronary pressure-flow mismatch are abundant in the literature 14, 15, 16, 17, 18, 19 and date back to the very earliest days of coronary physiological assessment (20). However, the observations made in our study provide new evidence demonstrating that in angiographically intermediate stenoses classified as FFR positive but iFR negative, the flow characteristics are similar to angiographically unobstructed vessels. Furthermore, in stenoses classified as FFR negative but iFR positive, the flow characteristics are similar to FFR+/iFR+ concordantly positive cases.

Within this study cohort, these findings suggest that when FFR/iFR discordance occurs, the true hyperemic flow-limiting potential of a stenosis is more accurately discernable by the iFR rather than the FFR measurement. Although iFR categorization in isolation cannot be used to fully determine coronary flow characteristics, in cases of FFR/iFR discordance, the FFR categorization is inversely related to hyperemic flow velocity, CFR, and the prevalence of diabetes. In the FFR–/iFR+ discordant group, the association of low CFR and high prevalence of diabetes may reflect the attenuating influence of microvascular disease on adenosine-mediated vasodilatation. Conversely, in the FFR+/iFR– discordant group, the association of high CFR and low prevalence of diabetes may reflect the effect of profound adenosine-mediated vasodilatation in healthy microcirculations.

### Discordance in stenosis classification by FFR and iFR: Clinical perspectives and implications

The present study provides physiological observations that can be useful to interpret the result of large clinical trials comparing iFR and FFR. In the RESOLVE (Multicenter core laboratory comparison of the instantaneous wave-free ratio and resting Pd/Pa with fractional flow reserve) study (3), FFR and iFR disagreed on the functional significance of an epicardial stenosis in approximately 20% of cases (3). More recently, Kobayashi et al. (21) reported that discordance between FFR and iFR was observed more frequently in left main or proximal left anterior descending artery lesions compared with other lesions. Therefore, discordance between hyperemic and resting indices is a common and important clinical finding, particularly as it occurs most frequently in vessels with the largest myocardial territories at stake.

The DEFINE-FLAIR (4) and iFR-SWEDEHEART (5) studies demonstrated in over 4,500 patients that iFR was noninferior to revascularization guided by FFR with respect to MACE at 1 year. Based on these 2 studies and the demonstrated quicker procedure time and decreased incidence of unpleasant patient side effects, iFR has recently been proposed as the preferred pressure-based index for the assessment of angiographically intermediate severity, stable coronary lesions (22). A further observation from the trials was that despite significantly less revascularization being performed based on iFR versus FFR interrogation, similar major adverse cardiac event rates were demonstrated in both FFR and iFR deferred populations (6). The findings of the present study do not extend to direct predictions of patient outcome, but do provide a possible mechanism to explain the higher revascularization rate associated with FFR.

### Study limitations

In this study, discordance was identified by differences in functional classification determined according to a single binary cut point value. Although myocardial ischemia must surely be a continuum, the use of binary cutpoints to distinguish hemodynamic significance from nonsignificance is ubiquitous in the literature, clinical outcome trials 4, 5, 9, 23, and revascularization and appropriate use criteria guidelines 24, 25, 26. This largely reflects the necessary design of clinical outcome trials, where revascularization decision-making must be standardized according to binary values. However, in clinical practice, the strict use of cutpoints may not be most appropriate.

The total number of discordant stenoses from the IDEAL study was relatively small. However, the IDEAL study represents the largest collection of patients with coronary artery disease undergoing physiological lesion assessment by combined pressure-and-flow measurements. The requirements for statistical analysis for differences between hyperemic flow velocity and CFR between groups were satisfied by the sample size. However, a larger number of discordant lesions may have permitted additional statistical power to determine if vessel type or stenosis location influences discordance between FFR and iFR (as has been demonstrated in larger [pressure-only] datasets) (21).

In the FFR+/iFR– discordant group, the median FFR was 0.77 (IQR: 0.74 to 0.80). Some readers may consider these to represent “gray zone” FFR values. Although no gray zone is incorporated into coronary revascularization guidelines 25, 26, clinicians do often apply a diagnostic gray zone in their practice in order to provide individualized patient decision-making. In such circumstances, readers may contest that additional information is required for FFR values of 0.75 to 0.80 to be considered truly flow limiting. In that regard, the direct measurement of intracoronary flow has been advocated (27), or as this study demonstrates, in cases of FFR/iFR discordance, the iFR classification alone appears able to accurately determine the hyperemic flow-limiting potential of a coronary stenosis.

In this study, CFR was used as the reference method for the determination of flow limitation of an angiographically intermediate coronary stenosis (Central Illustration). Although many consider CFR ≤2 to be indicative of myocardial ischemia, there is no universal normal value for CFR. Whether this level of CFR is adequate for some patients who still have ischemic responses despite CFR >2 remains a possibility. Mindful of these limitations to the use of CFR as a reference method, the inclusion of an unobstructed vessel group provides a clinically meaningful comparator of normality, given that the angiographic appearance of a vessel during coronary angiography is the first step in the clinical decision making process for the identification of ischemia (with a view to percutaneous coronary intervention). Furthermore, any potential criticism of using a ratio of coronary flow velocities to determine flow limitation are not founded in this dataset, as baseline flow velocities across all groups were comparable, including the unobstructed vessel group. Lastly, despite the angiographic lack of stenosis, 38 of 201 (19%) unobstructed vessels demonstrated the physiological pattern of negative FFR (>0.80) and low CFR (<2), likely indicative of ischemia caused by microvascular dysfunction. To provide a reference group that can be considered both angiographically and physiologically normal, a further analysis of unobstructed vessels with normal microvascular function is presented in Online Figure 1.

Because of a lack of clinical outcome data supporting its use, the whole-cycle Pd/aortic pressure (Pa) index was not included in this analysis. However, readers may wish to appreciate the relationship between iFR/Pd/Pa discordant cases as compared with CFR. In such circumstances, only the iFR categorization maintained a congruent relationship with CFR, irrespective of the cutoff value chosen for Pd/Pa (Online Figures 2 to 4). This finding further differentiates Pd/Pa and iFR, in addition to the differences already demonstrated with regard to lower iFR pressure-wire drift induced stenosis misclassification (8) and iFR virtual PCI capability (28).

Stenosed and unobstructed vessels were determined visually as per the original IDEAL study. Formal quantitative coronary angiography analysis was only performed post hoc. Therefore, operators may have visually overestimated stenosis severity as compared with the post hoc quantitative coronary angiography quantification of stenosis diameter (Table 4).

Last, in keeping with a previous large-scale study of discordance between hyperemic and resting pressure indices (16), the statistical unit of our analysis was vessels rather than patients. Accordingly, there is a potential for both statistical and biological interaction for different vessels analyzed within the same patient. However, across both the FFR–/iFR+ and FFR+/iFR– discordant groups, all but 4 vessels were from individual patients, and no patient contributed more than 1 vessel to both discordant groups. Indeed, repeat analysis after removal of discordant vessels from within the same patient did not alter the overall study findings (Online Figures 5 and 6). To permit a per-patient analysis, patients with more than 1 stenosis would need to be excluded, or alternatively, only 1 of the vessels selected for analysis. Either measure might risk the introduction of bias as well as limit the power of the study. Furthermore, an analysis of only 1 vessel per patient does not reflect real-world experience, where treating physicians make revascularization decisions on the vessel rather than patient level.

## Conclusions

In this analysis to determine the coronary flow characteristics of angiographically intermediate stenoses classified as discordant by FFR and iFR, discordance could be rationalized by differences in hyperemic coronary flow velocity, CFR and the prevalence of diabetes. Specifically, in comparison to FFR–/iFR+ discordant cases, FFR+/iFR– discordant cases were associated with higher hyperemic coronary flow velocity and CFR, and a lower prevalence of diabetes.

Additionally, coronary stenoses discordantly classified as FFR+/iFR– demonstrated similar coronary flow characteristics compared with angiographically unobstructed vessels. Although this observation does not extend to direct predictions of patient outcomes, it does provide some mechanistic insight helpful to interpreting the results of the recent large clinical trials 4, 5, where despite 5% fewer revascularizations performed with iFR, outcomes in both iFR and FFR deferred populations remained similar.

Perspectives **WHAT IS KNOWN?** Recent large clinical trials comparing iFR- and FFR-based revascularization decision making have demonstrated that despite significantly less revascularization being performed based on iFR versus FFR interrogation, similar MACE rates were demonstrated in both FFR and iFR deferred populations. The mechanism for this remains unclear.

**WHAT IS NEW?** In cases where FFR and iFR disagree, the iFR classification is more closely related to hyperemic coronary flow velocity (and CFR). Furthermore, the novel finding that coronary stenoses classified as FFR+/iFR– demonstrate similar coronary flow characteristics to unobstructed vessels indicates that FFR may overestimate the flow-limiting potential of angiographically intermediate coronary stenoses. Although this finding does not extend to direct predictions of patient outcomes, this latter observation provides mechanistic insight helpful to interpreting the results of the recent large clinical trials comparing iFR and FFR.

**WHAT IS NEXT?** In this analysis, measurements of Doppler-derived coronary flow were used as the gold standard test to determine physiological stenosis severity. Future patient outcome studies focused on FFR/iFR discordance will permit more definitive conclusions to be made.

## References

[1] Pijls N.H., Son JA van, Kirkeeide R.L., Bruyne B.D., Gould K.L. (1993). Experimental basis of determining maximum coronary, myocardial, and collateral blood flow by pressure measurements for assessing functional stenosis severity before and after percutaneous transluminal coronary angioplasty. Circulation.

[2] Sen S., Escaned J., Malik I.S. (2012). Development and validation of a new adenosine-independent index of stenosis severity from coronary wave-intensity analysis: results of the ADVISE (ADenosine Vasodilator Independent Stenosis Evaluation) study. J Am Coll Cardiol.

[3] Jeremias A., Maehara A., Généreux P. (2014). Multicenter core laboratory comparison of the instantaneous wave-free ratio and resting Pd/Pa with fractional flow reserve: the RESOLVE study. J Am Coll Cardiol.

[4] Davies J.E., Sen S., Dehbi H.-M. (2017). Use of the instantaneous wave-free ratio or fractional flow reserve in PCI. N Engl J Med.

[5] Götberg M., Christiansen E.H., Gudmundsdottir I.J. (2017). Instantaneous wave-free ratio versus fractional flow reserve to guide PCI. N Engl J Med.

[6] Escaned J. Safety of coronary revascularisation deferral based on iFR and FFR measurements in stable angina and acute coronary syndromes. Available at: http://solaci.org/_files/PCR2017/EscanedJavier.pdf. Accessed June 1, 2017.

[7] Nijjer S.S., Waard GA de, Sen S. (2016). Coronary pressure and flow relationships in humans: phasic analysis of normal and pathological vessels and the implications for stenosis assessment: a report from the Iberian–Dutch–English (IDEAL) collaborators. Eur Heart J.

[8] Cook C.M., Ahmad Y., Shun-Shin M.J. (2016). Quantification of the effect of pressure wire drift on the diagnostic performance of fractional flow reserve, instantaneous wave-free ratio, and whole-cycle Pd/Pa. Circ Cardiovasc Interv.

[9] Tonino P.A.L., De Bruyne B., Pijls N.H.J. (2009). Fractional flow reserve versus angiography for guiding percutaneous coronary intervention. N Engl J Med.

[10] Götberg M., Christiansen E.H., Gudmundsdottir I. (2015). Instantaneous Wave-Free Ratio versus Fractional Flow Reserve guided intervention (iFR-SWEDEHEART): rationale and design of a multicenter, prospective, registry-based randomized clinical trial. Am Heart J.

[11] Johnson N.P., Gould K.L. (2011). Physiological basis for angina and ST-segment change PET-verified thresholds of quantitative stress myocardial perfusion and coronary flow reserve. J Am Coll Cardiol Img.

[12] Johnson N.P., Gould K.L. (2012). Integrating noninvasive absolute flow, coronary flow reserve, and ischemic thresholds into a comprehensive map of physiological severity. J Am Coll Cardiol Img.

[13] Young D.F., Cholvin N.R., Kirkeeide R.L., Roth A.C. (1977). Hemodynamics of arterial stenoses at elevated flow rates. Circ Res.

[14] Akasaka T., Yamamuro A., Kamiyama N. (2003). Assessment of coronary flow reserve by coronary pressure measurement: comparison with flow- or velocity-derived coronary flow reserve. J Am Coll Cardiol.

[15] MacCarthy P., Berger A., Manoharan G. (2005). Pressure-derived measurement of coronary flow reserve. J Am Coll Cardiol.

[16] Echavarría-Pinto M., van de Hoef T.P., van Lavieren M.A. (2015). Combining baseline distal-to-aortic pressure ratio and fractional flow reserve in the assessment of coronary stenosis severity. J Am Coll Cardiol Intv.

[17] Petraco R., Hoef TP van de, Nijjer S. (2014). Baseline instantaneous wave-free ratio as a pressure-only estimation of underlying coronary flow reserve results of the JUSTIFY-CFR study (Joined Coronary Pressure and Flow Analysis to Determine Diagnostic Characteristics of Basal and Hyperemic Indices of Functional Lesion Severity–Coronary Flow Reserve). Circ Cardiovasc Interv.

[18] Johnson N.P., Kirkeeide R.L., Gould K.L. (2012). Is Discordance of coronary flow reserve and fractional flow reserve due to methodology or clinically relevant coronary pathophysiology?. J Am Coll Cardiol Img.

[19] Schelbert H.R. (2012). FFR and coronary flow reserve: friends or foes?. J Am Coll Cardiol Img.

[20] Serruys P.W., Di Mario C., Meneveau N. (1993). Intracoronary pressure and flow velocity with sensor-tip guidewires: A new methodologic approach for assessment of coronary hemodynamics before and after coronary interventions. Am J Cardiol.

[21] Kobayashi Y., Johnson N.P., Berry C. (2016). The influence of lesion location on the diagnostic accuracy of adenosine-free coronary pressure wire measurements. J Am Coll Cardiol Intv.

[22] Bhatt D.L. (2017). Assessment of stable coronary lesions. N Engl J Med.

[23] De Bruyne B., Fearon W.F., Pijls N.H.J. (2014). Fractional flow reserve–guided PCI for stable coronary artery disease. N Engl J Med.

[24] Patel M.R., Dehmer G.J., Hirshfeld J.W. (2012). ACCF/SCAI/STS/AATS/AHA/ASNC/HFSA/SCCT 2012 appropriate use criteria for coronary revascularization focused update. J Thorac Cardiovasc Surg.

[25] Windecker S., Kolh P. (2014). 2014 ESC/EACTS Guidelines on myocardial revascularization: The Task Force on Myocardial Revascularization of the European Society of Cardiology (ESC) and the European Association for Cardio-Thoracic Surgery (EACTS) * Developed with the special contribution of the European Association of Percutaneous Cardiovascular Interventions (EAPCI). Eur Heart J.

[26] Fihn S.D., Gardin J.M., Abrams J. (2012). 2012 ACCF/AHA/ACP/AATS/PCNA/SCAI/STS Guideline for the diagnosis and management of patients with stable ischemic heart disease: a report of the American College of Cardiology Foundation/American Heart Association Task Force on Practice Guidelines, and the American College of Physicians, American Association for Thoracic Surgery, Preventive Cardiovascular Nurses Association, Society for Cardiovascular Angiography and Interventions, and Society of Thoracic Surgeons. J Am Coll Cardiol.

[27] Hoef TP van de, Siebes M., Spaan J.A.E., Piek J.J. (2015). Fundamentals in clinical coronary physiology: why coronary flow is more important than coronary pressure. Eur Heart J.

[28] Götberg M., Cook C.M., Sen S., Nijjer S., Escaned J., Davies J.E. (2017). The evolving future of instantaneous wave-free ratio and fractional flow reserve. J Am Coll Cardiol.

