# Supplementary material for: Fractional Flow Reserve/Instantaneous Wave-Free Ratio Discordance in Angiographically Intermediate Coronary Stenoses: An Analysis Using Doppler-Derived Coronary Flow Measurements
Source: JACC Cardiovasc Interv. 2017 Dec 26;10(24):2514–24. doi: 10.1016/j.jcin.2017.09.021 (PMC5743106; doi:10.1016/j.jcin.2017.09.021)
Supplement: Online Figures 1–6 [file mmc1.docx]

Online figure 1

Online figure 2

Online figure 3

Online figure 4

Online figure 5

Online figure 6
